# Development of a user guide to support administration of the MRC Prion Disease Rating Scale in research and clinical settings for prion diseases

**DOI:** 10.1080/19336896.2025.2565204

**Published:** 2025-09-30

**Authors:** Robert S. Pulido, Chris Marshall, Anne V. Smith, Hannah Edge, Aaron Yarlas, Brian Appleby, Jean-Philippe Brandel, Steven Collins, Nurit Omer, Inga Zerr, Simon Mead

**Affiliations:** a Ionis, Carlsbad, CA, USA; b Clinical Outcomes Assessment, Clarivate, London, UK; c National Prion Disease Pathology Surveillance Center, Case Western Reserve University, University Hospitals Cleveland Medical Center, Cleveland, OH, USA; d Cellule Nationale de Référence des Maladies de Creutzfeldt-Jakob, Institut du Cerveau et de la Moelle épinière (ICM), Sorbonne Université, INSERM, CNRS UMR 7225, AP-HP, University Hospital Pitié-Salpêtrière, Paris, France; e Australian National Creutzfeldt-Jakob Disease Registry, The Florey Institute of Neuroscience and Mental Health and Department of Medicine (RMH), The University of Melbourne, Parkville, Australia; f The Neurological Institute, Tel Aviv Sourasky Medical Center, Tel Aviv, Israel; g National Reference Center for Human Prion Diseases, Department of Neurology, University Medical Center, Göttingen, Germany; h Institute of Prion Diseases, MRC Prion Unit at University College London, London, UK

**Keywords:** CJD, outcome measure, prion, rating scale

## Abstract

**Background:**

The prion diseases (PrD) are a group of progressive, fatal, neurodegenerative diseases, for which the Medical Research Council Prion Disease Rating Scale (MRC Scale) can be used to assess patients’ functional deterioration. Findings from previous qualitative interviews with caregivers and clinical experts identified potential ambiguities in the scale that could lead to inconsistent scoring within and/or between raters.

**Methods:**

A draft User Guide was developed based on findings from a previous qualitative study. The draft included clarifications regarding domain wording, scoring levels, and guidance for response option selection. Five clinical experts with PrD management experience provided written feedback on the draft User Guide, which was incorporated into a revised User Guide. A 90-minute consensus meeting was then held with these experts to confirm the final content to be included in the User Guide.

**Results:**

The final User Guide was designed to accompany the MRC Scale and assist with rater decisions related to which response option most accurately describes a patient’s health status. Conclusions: The User Guide is expected to be a valuable complement to the MRC Scale, which is poised to rise in use and prominence as global clinical research efforts accelerate to address the significant unmet need of PrD patients.

## Introduction

The prion diseases (PrD) are a group of rare, progressive, fatal, neurodegenerative diseases, with an incidence of 1–2 cases per million [1]. Disease onset varies but is typically between 50 and 70 years old; 80–90% die within one year of symptom onset [2]. Common symptoms of PrD include cognitive impairments (e.g., memory, language, judgement), involuntary muscle spasms (myoclonus) and loss of coordination (cerebellar ataxia) [3,4]. Functional deterioration can occur rapidly in patients with PrD, meaning that they can reach an akinetic mutism state within several months of disease onset [5], and death usually occurs at a median of 4–6 months after onset of illness in patients with Creutzfeldt-Jakob disease (CJD), the most common PrD [6].

The PrD field is at an exciting stage, with increasingly global research underway to characterize the natural history of the diseases and to evaluate potential disease-modifying treatments [7]. Effective treatments would change the landscape of PrD clinical care [7]. To detect potential benefits of these treatments on patients’ health, there will be a need for simple tools that readily and reliably capture a patient’s clinical condition over time. The Medical Research Council Prion Disease Rating Scale (MRC Scale hereafter) is well positioned to serve this need, and its use can be expected to increase globally in both research settings and clinical care as investigational treatments are tested, and effective treatments emerge. The development of the MRC Scale began after analysis of outcome measures in the UK’s PRION-1 trial showed that survival and many generic cognitive scales were suboptimal outcome measures for PrD, whereas functionally oriented measures such as the Barthel Index and CDR-SB offered better validity and statistical power for trials [8]. Building on these findings, the UK’s National Prion Monitoring Cohort study combined items from Barthel, CDR-SB and the Glasgow Coma Score with caregiver-prioritized domains and refined them using Rasch modelling to produce the 20-point MRC Scale with minimal floor effects and reliability for in-person or telephone administration [9]. The MRC Scale was then validated across prospective cohorts, supporting its use as a pragmatic, functionally oriented outcome measure for clinical trials and longitudinal care [10–14]

The MRC Scale is used to assess the functional deterioration in a patient with PrD from the clinician’s perspective through interviewing the caregiver and patient (to the extent that input from the patient is possible) [9] and thereby disease progression through serial assessments [9,11,13,15]. The MRC Scale may be completed during a clinical study or during routine care visits. Although strong inter-rater reliability has been demonstrated [9], efforts to further improve standardization of MRC Scale administration would help to ensure that it is a robust and reproducible tool to measure PrD severity and progression.

**Figure 1.**
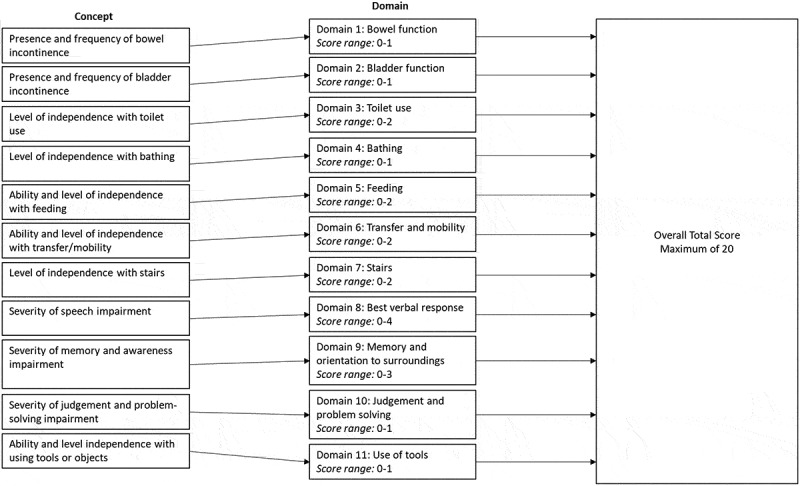
Conceptual framework for the MRC Scale.

The conceptual framework of the MRC Scale originated from the analysis of the UK National Prion Monitoring Cohort and was adapted based on interviews with caregivers to identify the most clinically relevant manifestations of PrD. The final version of the MRC Scale comprises 11 items. Each item measures a distinct functional domain, which is summed to produce an overall total score (Figure 1).

A cross-sectional, non-interventional, qualitative interview study was conducted to generate evidence to support the content validity of the MRC Scale. The study comprised combined concept elicitation and cognitive-debriefing interviews with 12 informal former caregivers (i.e., individuals who provided unpaid and ongoing assistance, often spouses or children) of patients with PrD and six clinical experts (including the senior co-developer of the MRC Scale [SM]) with experience of assessing/treating patients with PrD. Four of these six clinical experts were familiar with and had used the scale in research and/or practice settings. Findings from these interviews supported the MRC Scale as a conceptually relevant measure, with each of the 11 assessed domains consistently mentioned by both caregivers and clinicians as representing important aspects of the functional patient capacity in PrD. Response options were felt to reflect different clinical states with meaningful scoring gradation. However, ambiguities were identified during the cognitive debriefing sections of the interviews with clinical experts that could result in inconsistent scoring of patients across raters (e.g., response option ambiguity).

FDA regulatory guidance states that clinician-reported outcomes (ClinROs) such as the MRC Scale should *‘include a user manual with clear instructions and directions for standardized administration’* [16]. To address these perceived ambiguities and facilitate consistent administration and scoring, the development of a detailed User Guide was deemed valuable to support reliable scoring between and within raters.

## Materials and methods

**Figure 2.**
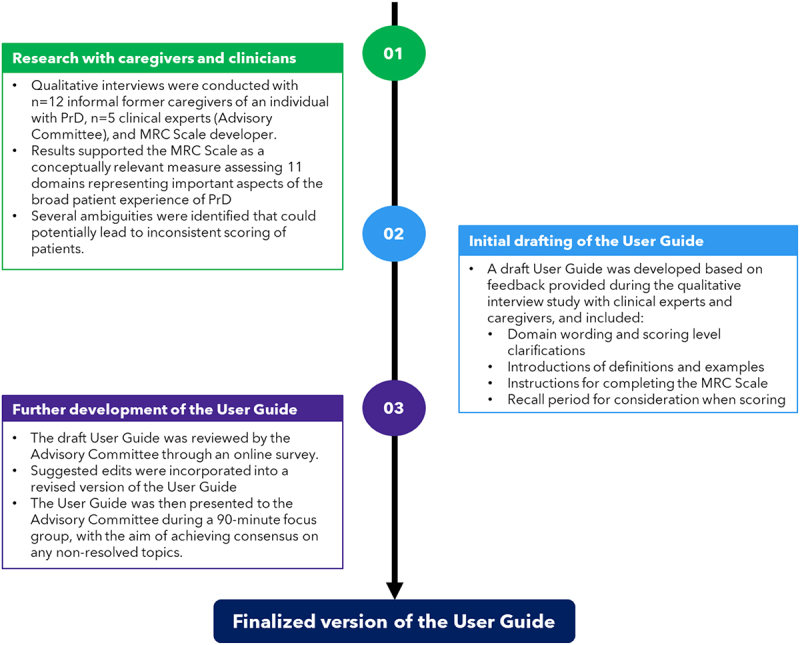
Methods used to develop a user guide for the MRC Scale.

An overview of the methods used to develop the User Guide is presented in Figure 2.

**Stage 1**: Interviews for the initial qualitative study conducted with 12 caregivers and, separately, with 6 clinicians (including a senior co-developer of MRC Scale [SM]) lasted approximately 45 minutes each and were conducted remotely. Caregivers and clinicians provided written informed consent prior to study participation. A key focus of the clinical expert interviews was to determine which questions or response options could have greater clarification and would benefit from formal guidance in an MRC Scale user guide.

**Stage 2**: After completion of Stage 1, a draft User Guide was then developed to incorporate feedback provided during the qualitative interview study with clinical experts and caregivers.

**Stage 3**: Clinicians were re-consented prior to participation in the second study. The draft User Guide, along with an online survey containing 70 questions (including both closed and open-style questions), was sent to five clinical experts with significant experience in the assessment and management of PrD (referred to here as the Advisory Committee, who are included as co-authors of this paper: BA, J-PB, SC, NO, and IZ). All five members of the Advisory Committee had previously participated in the qualitative interview study described in Stage 1. Questions that received ≥80% consensus were considered to be resolved and were not planned for discussion during the consensus meeting. Questions that received <80% consensus (i.e., lack of agreement among at least 4 of the 5 members of the Advisory Committee) during the survey were noted as points to be further discussed during the subsequent consensus meeting. Any other emergent topics, such as recall period and causes of impairment, were also marked for follow-up discussion. The revised draft of the User Guide was then presented to the Advisory Committee during a 90-minute focus group teleconference meeting with the aim of achieving, through discussion, consensus on any remaining ambiguities raised during the online survey.

## Results

**Stage 1**: Twelve informal former caregivers of individuals with PrD participated in Stage 1 interviews. Most of the caregivers were female (*n* = 8), and in a spousal relationship with their care recipient (*n* = 11), with a mean age of 59 years old (ranging from 34 to 75 years old). All five clinical experts (excluding the senior co-developer) involved in Stages 1–3 were neurologists or neuropsychiatrists, with a mean time of treating patients with PrD of 22.8 years, ranging from 10 to 30 years. Each clinical expert was from a different country, including Australia, France, Germany, Israel, and the United States. All caregivers reported that a majority of the domains and response options were relevant to their experience of PrD. Some caregivers had suggestions for additional domains that were relevant to their individual experience with PrD (e.g., behavioural changes, emotional well-being). During an interview with the MRC Scale senior co-developer, he explained that items that might have responded to symptomatic therapy and/or would not be expected to progress in tandem with overall disease progression were excluded from measure development and could instead be assessed using other tools. Overall, all caregivers agreed that the MRC Scale sufficiently covers the most important functional aspects of PrD.

The clinical experts confirmed that patients with PrD experience dysfunction or decline in all of these domains on the MRC Scale. Generally, the clinical experts found the response options to be appropriate and clinically discrete. The experts provided useful feedback to promote consistent scoring across raters, as summarized below.

1. Clear definitions were requested for some words, including ‘occasional’ (Bladder function domain), ‘help’ (Toilet use, Bathing, Feeding, Transfer and mobility, and Stairs domains), ‘some impairment on and off’ (Memory and orientation to surroundings domain) and ‘severely impaired’ (Judgement and problem solving domain).
2. Guidance was requested regarding the conceptual coverage of each domain. Specifically: Should the Bowel function and Bladder function domain scores consider only lack of bowel/bladder control or also consider episodes of incontinence due to mobilization issues (e.g., not reaching the toilet), cognitive issues (e.g., not realizing the need to toilet), or laxative use? Should the Toilet Use domain score consider only activities using the toilet, or also mobilization issues (e.g., getting to the toilet)? Should the Bathing domain score consider only mobility issues or also consider bathing issues caused by cognitive impairments? Should the Feeding domain score consider only swallowing difficulties or also consider cognitive impairments (e.g., needing to be reminded to eat) or motor impairments (e.g., difficulty physically cutting up food and getting it to the mouth)? Should the Best verbal response domain include moaning? Should the Tools domain score consider difficulty using tools due to cognitive impairment, motor impairment or both?
3. For the Bathing domain, clarification was requested regarding whether to include the patient’s ability to shower.
4. For the Stairs domain, clarification was requested regarding how to rate patients who do not often encounter stairs, such as those who live in single-story houses.

**Stage 2**: The draft User Guide included the requested clarifications regarding domain wording (such as what is meant by ‘help’), scoring levels, and selection of response options. General instructions for completion of the MRC Scale and a recall period for raters to consider when selecting scores for each domain were also included in the initial draft User Guide. The senior co-developer of the MRC Scale provided guidance on the intended use and scoring of domains (e.g., the Toilet use domain was intended to include consideration of complex motor tasks like personal hygiene and clothing removal, which differentiates it from the Incontinence domain). Additional clarifications included the intended definition of ‘tools’ as items such as a knife and fork, a cup, and a remote control. The senior co-developer was supportive of development of a User Guide to accompany the MRC Scale.

**Stage 3**: Of the 70 questions included in the online survey, 41 reached consensuses during the survey, 21 questions did not reach consensus during the survey, and eight questions were not phrased to gain consensus (i.e., did not have ‘yes/no’ or ‘agree/disagree’ response options). Comments and proposed amendments based on the Advisory Committee’s feedback collated during the online survey were summarized and edits based on consensual feedback were incorporated into a revised draft of the User Guide. These edits included establishing a recommended recall period for the MRC Scale, discussing the topic of clarifying the causes of impairment, the inclusion and selection of examples of ‘help’ and ‘devices’ for certain domains, clarifying what is meant by ‘independence’ in terms of the need for ‘help’/’device’ usage, and general rephrasing of some aspects of the guidance. During the consensus meeting, all changes that had been made based on the survey feedback were confirmed as appropriate. The instructional text for each of the 11 domains was deemed by the Advisory Committee to be supportive of consistent and standardized administration and domain scoring across raters. Consensus was also reached on an appropriate recall period for domains 2–11 (‘past 24 hours’) and that cause of impairment (e.g., whether cognitive or motor) should not be considered when selecting a score for each domain. Three minor edits were made to the User Guide based on the consensus meeting discussion (wording of recall period, and additional clarification to the Bathing and Transfer & Mobility domains).

The final User Guide (see supplementary material) was designed to help the rater decide, for each domain, which response option most accurately describes a patient’s functional and cognitive status. The User Guide developed in this study was designed to accompany and complement the MRC Scale; no changes were made to the scale itself during this process.

## Discussion

The MRC Scale is a validated instrument designed to capture the functional status of individuals with PrD, which has shown good content validity in qualitative interviews with caregivers and experts and good psychometric properties across several studies [9,12,17,18]. Following a robust development process involving five clinical experts with significant experience in the diagnosis and management of PrD, an MRC Scale User Guide (open access) has been developed to support consistent and standardized administration and domain scoring across and within raters. The User Guide provides advice to mitigate potential ambiguities and prevent differences in interpretation of the language used in the MRC Scale (e.g., specifying a recall period, disregarding cause of impairment, providing examples to clarify context for scoring). The User Guide is expected to be a valuable complement to the MRC Scale, supporting its standardized use and interpretability as global clinical research efforts advance to address the significant unmet need of PrD patients.

## Ethics

This study was performed in accordance with the Declaration of Helsinki. The study protocol and associated documents for the initial qualitative interview study with caregivers were submitted for review to the Western Copernicus Group Institutional Review Board (WCG IRB) and received approval on 20 July 2022 (IRB Study Number: 1336553). The WCG IRB was selected to perform the ethical review of the initial qualitative interview study as they are registered with the Office for Human Research Protections and are compliant with the Food and Drug Administration (FDA). Written informed consent was obtained from both caregivers and clinical experts prior to the study.

## Supplementary Material

10711_MRCRatingScale_UserGuide_Supplementary material.doc

## Data Availability

The final copy of the User Guide is available within the supplementary materials.
