# Supplementary material for: Development of a user guide to support administration of the MRC Prion Disease Rating Scale in research and clinical settings for prion diseases
Source: Prion. 2025 Sep 30;19(1):1–7. doi: 10.1080/19336896.2025.2565204 (PMC12489992; doi:10.1080/19336896.2025.2565204)
Supplement: 10711_MRCRatingScale_UserGuide_Supplementary material.doc [file KPRN_A_2565204_SM1551.doc]

The MRC Scale: User Guide

Contents

[Background of the MRC Scale 3](#__RefHeading___Toc190073223)

[Purposes of the User Guide 3](#__RefHeading___Toc190073224)

[Overall Guidance 3](#__RefHeading___Toc190073225)

[1. Bowel Function 4](#__RefHeading___Toc190073226)

[2. Bladder Function 4](#__RefHeading___Toc190073227)

[3. Toilet Use 4](#__RefHeading___Toc190073228)

[4. Bathing 5](#__RefHeading___Toc190073229)

[5. Feeding 5](#__RefHeading___Toc190073230)

[6. Transfer and Mobility 6](#__RefHeading___Toc190073231)

[7. Stairs 6](#__RefHeading___Toc190073232)

[8. Best verbal response 7](#__RefHeading___Toc190073233)

[9. Memory and orientation to surroundings 7](#__RefHeading___Toc190073234)

[10. Judgement and problem solving 8](#__RefHeading___Toc190073235)

[11. Use of tools 8](#__RefHeading___Toc190073236)

[References 9](#__RefHeading___Toc190073237)

Background of the MRC Scale

The MRC Prion Disease Rating Scale (MRC Scale for short) is a functionally-oriented 20-point scale that assesses 11 functional domains in prion disease patients, including cognitive function, speech, mobility, personal care/feeding and continence, according to their relative importance documented by carer interviews in previous research (Thompson et al., 2013). Higher scores indicate a better functional state (domain and total score). The MRC Scale can be administered remotely or in person.

Scale development involved modifying certain elements of three rating scales – the Modified Barthel Activities of Daily Living Index (Mahoney and Barthel, 1965), the memory, orientation and judgement/problem-solving subcomponents of the Clinical Dementia Rating Sum of Boxes (Morris, 1993), the best verbal response subcomponent of the Glasgow Coma Score (Teasdale and Jennett, 1974) – and combining them with a novel assessment of the ability to use tools. Minor modifications were made to subcomponents to make them more suitable for use in patients at all stages of prion disease (Thompson et al., 2013), e.g., patients who are experiencing mutism.

Semi-quantitative and qualitative interviews were conducted with patients’ relatives and carers, and the Barthel Activities of Daily Living Caregiver Interview Summary form (Mahoney and Barthel, 1965) was used to gather data on which manifestations of prion disease were of greatest concern to them to ensure that they were reflected in the domains assessed by the scale (Thompson et al., 2013). Scale development also involved item response modelling (Rasch analysis) (Hobart et al., 2007), inter-rater reliability testing, construct analysis and correlation with several other existing scales to refine the outcome measure for use in a prion disease clinical trial (Thompson et al., 2013).

Purposes of the User Guide

The purposes of this user guide are as follows:

To facilitate the consistent administration of the MRC Scale by providing guidance to support the
 standardization of scoring between raters and within raters.

To help the rater decide, for each domain, which response option most accurately describes a patient’s
 health status.

To facilitate consistent monitoring of disease progression, for example in clinical trials and other settings.

Guidance included within this manual is based on interviews with and advice from caregivers of prion disease patients and clinical experts in prion disease, including one of the experts who developed the MRC Scale.

Overall Guidance

Please use your best judgement to select one score for each question based on your discussions and interactions with the caregiver and the patient and guided by the instructional information provided for each domain.

For each domain, please select a response based on the patient’s current condition (what the patient was able to do in the past 24 hours), but this does not apply for Domain 1 (Bowel Function)”.

| 1. Bowel Function   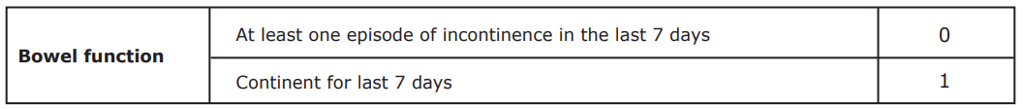  Consider incontinence due to **any** cause.  ‘Incontinence’ refers only to bowel incontinence.  For patients with any type of ostomy bag, please select “0”. |
| --- |

| 1. Bladder Function   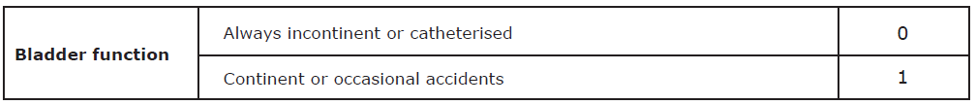  Consider incontinence due to **any** cause.  ‘Incontinence’ refers only to bladder incontinence.  If patient experiences incontinence every time they urinate, please select “0;” otherwise please select “1”. |
| --- |

| 1. Toilet Use   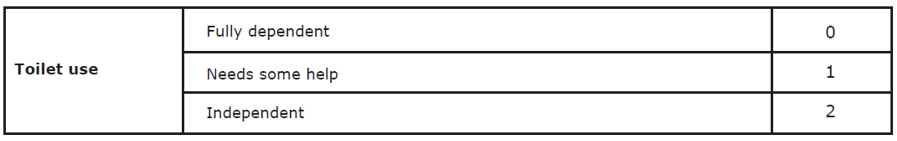  ‘Toilet use’ refers to the processes of toileting that take place within the bathroom, including, for example: manipulating clothing on/off, getting on/off the toilet, effectively using toilet paper.  ‘Help’ includes, for example: verbal prompts/reminders, physical assistance, and/or the need for use of a device (e.g., handrails, toilet seat lift, commode chair, urinary bottle, condom drainage catheter).  For patients who are catheterized and have any type of ostomy (i.e., do not use the toilet), please select “0”. |
| --- |

| 1. Bathing   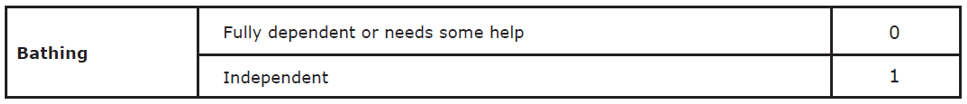  ‘Bathing’ includes use of a bath or a shower.  Consider all bathing activities that take place within the bathroom (e.g., manipulating clothing on/off, getting in/out the bath, opening bottles, effectively washing self).  ‘Help’ includes, for example, verbal prompts/reminders, physical assistance, and/or the need for use of a device (e.g., bath/shower chair, a bath/shower support rail and/or a hoist transfer).  If a patient can use a bath but not a shower, or vice versa, the patient should be scored ‘0’ |
| --- |

| 1. Feeding   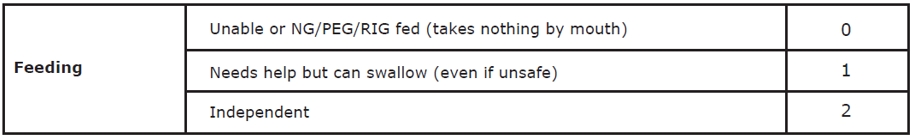  ‘Feeding’ refers to the full process of eating, including, for example, using a knife and fork to cut food, moving food to the mouth, chewing, and swallowing.  ‘Help’ includes, for example:  verbal prompts/reminders,  physical assistance with cutlery use (e.g. overlapping hands with the patient),  another person feeding the patient, and/or  the provision of pre-cut or pureed food. |
| --- |

| 1. Transfer and Mobility   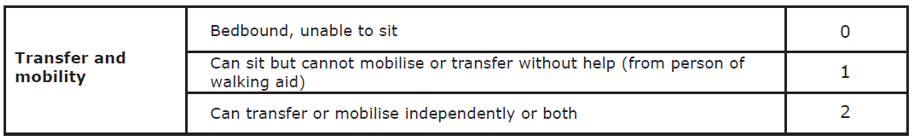  ‘Can sit’ is defined as independently maintaining appropriate balance and posture while sitting in a chair or wheelchair, without the use of straps or belts.  Consider mobilising on flat ground or a gentle incline only.  Consider the following transfer activities:  In/out of bed  On/off a chair  Getting on/off the toilet  ‘Help’ includes, for example, verbal prompts/reminders, physical assistance, and/or need for use of a device (e.g., cane, walker, wheelchair, support rail). |
| --- |

| 1. Stairs   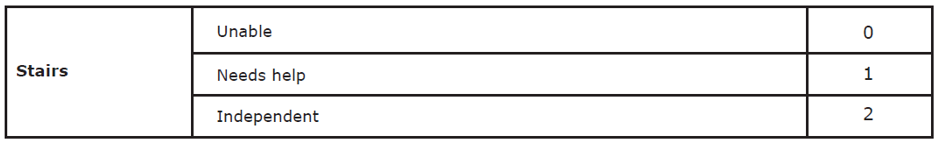  ‘Stairs’ are defined as a flight of multiple steps from one story of a building to another.  ‘Help’ includes, for example, verbal prompts/reminders, physical assistance including **need** for use of a handrail, and/or requiring accompaniment.  If no direct observation (e.g., patient has not recently encountered stairs), use your best judgment based on the patient’s current condition to select a score.  If the patient requires a chair lift to ascend or descend stairs, please select “0”. |
| --- |

| 1. Best verbal response   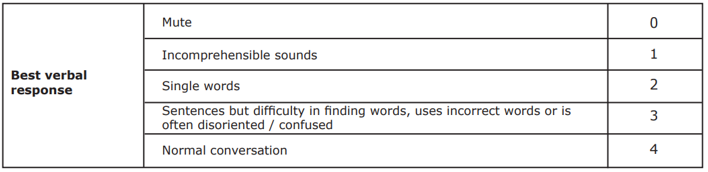  ‘Incomprehensible sounds’ include purposeful sounds (e.g., moaning or grunting that is expressed as a form of communication).  Incomprehensible sounds that are considered to be non-purposeful should not be considered when scoring. |
| --- |

| 1. Memory and orientation to surroundings   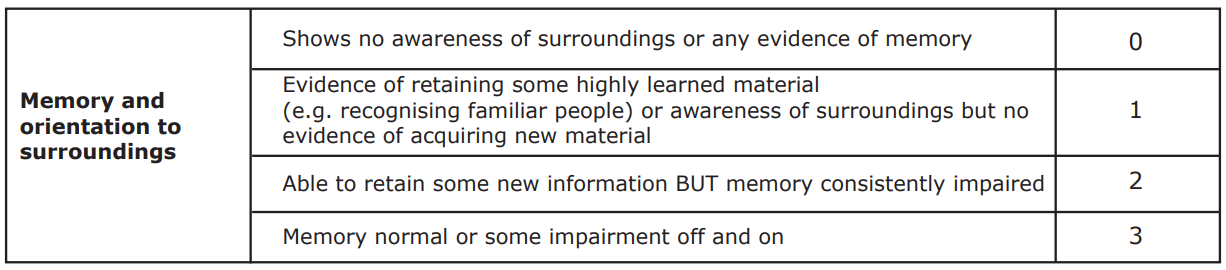  Consider non-verbal as well as verbal indications of memory/awareness (e.g., eyes widening at sight of loved ones by bedside).  ‘Some impairment off and on’ is defined as occasional lapses in memory (i.e., not consistent/persistent impairment). |
| --- |

| 1. Judgement and problem solving   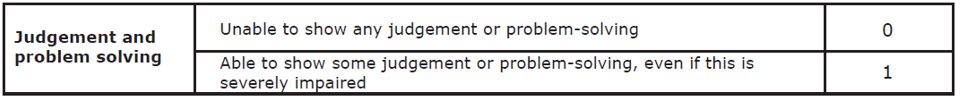  ‘Judgement and problem solving’ is defined as the ability to perform basic judgements.  Examples of ‘judgement or problem-solving’ include (but are not limited to):  Knowing how to perform daily tasks appropriately (e.g., preparing food, getting dressed and undressed, turning on/off an electrical device);  General decision making (e.g., completing forms appropriately, opening the door when someone knocks, answering the telephone when it rings).  Patients with normal functioning should be scored as ‘1’. |
| --- |

| 1. Use of tools   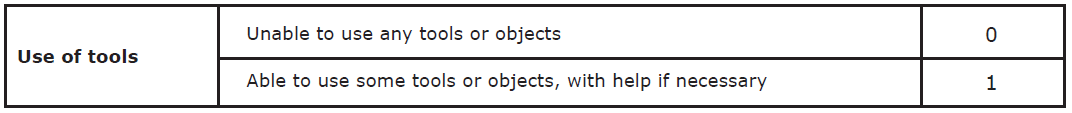  Tools are physical objects that are intended for a purposeful use, such as:  cutlery,  a remote control,  a pen or pencil,  a telephone/cell phone,  a toothbrush,  a hairbrush.  ‘Help’ includes, for example:  Verbal prompts/reminders, and/or  Partial physical assistance that does not fully replace the patient’s own performance.  Patients with normal functioning should be scored as ‘1’.  Patients who are able to use any tool with any level of effectiveness should be scored as ‘1’.  Patients who are not able to use any tool for any reason (e.g., physical, cognitive, and/or other disability) should be scored as ‘0’. |
| --- |

References

HOBART, J. C., CANO, S. J., ZAJICEK, J. P. & THOMPSON, A. J. 2007. Rating scales as outcome measures for clinical trials in neurology: problems, solutions, and recommendations. *The Lancet Neurology,* 6**,** 1094-1105.

MAHONEY, F. I. & BARTHEL, D. W. 1965. Functional Evaluation: The Barthel Index. *Md State Med J,* 14**,** 61-5.

MORRIS, J. C. 1993. The Clinical Dementia Rating (CDR): current version and scoring rules. *Neurology,* 43**,** 2412-4.

TEASDALE, G. & JENNETT, B. 1974. Assessment of coma and impaired consciousness. A practical scale. *Lancet,* 2**,** 81-4.

THOMPSON, A. G., LOWE, J., FOX, Z., LUKIC, A., PORTER, M. C., FORD, L., GORHAM, M., GOPALAKRISHNAN, G. S., RUDGE, P., WALKER, A. S., COLLINGE, J. & MEAD, S. 2013. The Medical Research Council prion disease rating scale: a new outcome measure for prion disease therapeutic trials developed and validated using systematic observational studies. *Brain,* 136**,** 1116-27.
